# Streptococcal Cysteine Protease-Mediated Cleavage of Desmogleins Is Involved in the Pathogenesis of Cutaneous Infection

**DOI:** 10.3389/fcimb.2018.00010

**Published:** 2018-01-24

**Authors:** Tomoko Sumitomo, Yasushi Mori, Yuumi Nakamura, Mariko Honda-Ogawa, Seitaro Nakagawa, Masaya Yamaguchi, Hiroyuki Matsue, Yutaka Terao, Masanobu Nakata, Shigetada Kawabata

**Affiliations:** ^1^Department of Oral and Molecular Microbiology, Osaka University Graduate School of Dentistry, Osaka, Japan; ^2^Division of Special Care Dentistry, Osaka University Dental Hospital, Osaka, Japan; ^3^Department of Dermatology, Chiba University Graduate School of Medicine, Chiba, Japan; ^4^Division of Microbiology and Infectious Diseases, Niigata University Graduate School of Medical and Dental Sciences, Niigata, Japan

**Keywords:** *Streptococcus pyogenes*, epidermal barrier, cutaneous infection, desmogleins, SpeB

## Abstract

*Streptococcus pyogenes* is responsible for a wide variety of cutaneous infections ranging from superficial impetigo to fulminant invasive necrotizing fasciitis. Dysfunction of desmosomes is associated with the pathogenesis of cutaneous diseases. We identified streptococcal pyrogenic exotoxin B (SpeB) as a proteolytic factor that cleaves the extracellular domains of desmoglein 1 and 3. In an epicutaneous infection model, lesional skin infected with an *speB* deletion mutant were significantly smaller as compared to those caused by the wild-type strain. Furthermore, immunohistological analysis indicated cleavage of desmogleins that developed around the invasion site of the wild-type strain. In contrast, the *speB* mutant was preferentially found on the epidermis surface layer. Taken together, our findings provide evidence that SpeB-mediated degradation of desmosomes has a pathogenic role in development of *S. pyogenes* cutaneous infection.

## Introduction

*Streptococcus pyogenes* causes a broad spectrum of cutaneous infections, ranging from superficial streptococcal pyoderma to moderately severe cellulitis and even life-threatening necrotizing fasciitis. The annual prevalence of streptococcus pyoderma is estimated to be more than 111 million cases worldwide, with economically disadvantaged children living in tropical and subtropical areas most frequently affected (Carapetis et al., 2005). Epidemiological data have shown an association with subsequent development of post-infectious glomerulonephritis and invasive diseases in patients with streptococcal pyoderma, which has important implications for their prognosis.

The epidermis provides the primary defense against pathogenic invaders. Its cutaneous barrier function is achieved by the existence of a cornified layer and firm adhesion between adjacent keratinocytes joined by a series of intercellular junctions, such as tight junctions (TJs), adherence junctions (AJs), and desmosomes (DMs). It is likely that intraepidermal invasion of *S. pyogenes* through sites of abrasions, minor trauma, or insect bites is required for development of superficial skin infection (Stevens and Bryant, 2016). We recently reported that *S. pyogenes* possesses several strategies for TJ and AJ destabilization allowing for bacterial penetration via a paracellular route, raising the possibility that disruption of the keratinocyte barrier by colonized *S. pyogenes* leads to pyoderma development (Sumitomo et al., 2011, 2013, 2016; Sumitomo, 2015).

Desmogleins, the major constituents of DMs, play an essential role in maintenance of the structure and barrier function of the epidermis. Two desmoglein isoforms are mainly expressed in human epidermis in a differentiation-dependent manner. One is desmoglein 1 (Dsg1) which is distributed through the spinous and granular layers, and the other is desmoglein 3 (Dsg3) which is predominantly expressed in the basal and immediate suprabasal layers. Although dysfunction of these DM components has been implicated to play a crucial role in the pathogenesis of skin diseases, such as pemphigus and bullous impetigo, little is known about the correlation between loss of cell-cell adhesion and clinical manifestation of streptococcal pyoderma (Whittock and Bower, 2003; Amagai, 2010).

In the present study, we obtained novel findings showing that an *S. pyogenes*-secreted protease, SpeB, cleaves the extracellular domains of Dsg1 and Dsg3. Furthermore, by use of a mouse epicutaneous infection model, we addressed the mechanism underlying the onset of superficial cutaneous infections caused by *S. pyogenes*. Our results revealed that *S. pyogenes* proteolytic activity impairs epidermal barrier function, thereby leading to bacterial invasion of intraepidermal space and formation of an ecthyma-like lesion.

## Materials and methods

### Bacterial strains and culture conditions

*S. pyogenes* clinical isolates, strains SSI-9 (serotype M1), SSI-1 (M3), 30 (M12), and NIH35 (M28), were isolated from patients with streptococcal toxic shock syndrome. Other clinical isolates, strains SF370 (serotype M1), TW3358 (M3), TW3337 (M12), TW3339 (M28), NZ131 (M49), and 591 (M49), were non-invasive *S. pyogenes* strains. All strains were cultured in Todd-Hewitt broth (Becton, Dickinson and Company; BD) supplemented with 0.2% yeast extract (BD) (THY medium) at 37°C in an ambient atmosphere. *Escherichia coli* strain BL21-CodonPlus (DE3)-RIPL (Agilent Technologies) was used as a host for the plasmids pQE30 and pREP4 (Qiagen). All *E. coli* strains were cultured in Luria-Bertani (Nacalai Tesque) (LB) medium at 37°C with agitation. For selection and maintenance of *E. coli* mutant strains, antibiotics were added to the medium at the following concentrations, including ampicillin (100 μg/ml), kanamycin (30 μg/ml), and chloramphenicol (34 μg/ml).

### Preparation of recombinant proteins and *S. pyogenes* mutant strains

An in-frame *speB* deletion mutant and its revertant strain with a background of NIH35 or 591 were constructed using the pSET4s temperature-sensitive shuttle vector, as previously reported (Takamatsu et al., 2001; Nakata et al., 2011; Sumitomo et al., 2011). Preparation of recombinant SpeB was performed as previously described (Terao et al., 2008). For construction of the recombinant proteins Dsg1 and Dsg3, cDNA from HaCaT cells was prepared using Trizol and a PureLink RNA mini-kit (Thermo Fisher Scientific). Next, cDNA fragments encoding the extracellular domain of Dsg1 or Dsg3 were amplified using the following specific primers: rdsg1F (5′- CGGGATCCGAATGGATCAAGTTCGCAGCAGCCTGTCG-3′), rdsg1R (5′- GGGGTACCATGCACATTGTCTGATAACAA-ATCTTTGGCTCCG-3′), rdsg3F (5′- CGGGATCCGAATGGG-TGAAATTTGCCAAACCCTGC-3′), and rdsg3R (5′- GGGG-TACCGCGGCCTGAGTGCGGCCTGCCATACCTGG-3′). The primers were designed using the reported mRNA sequences for *Homo sapiens* desmoglein-1 (GenBank accession number Q02413) and *Homo sapiens* desmoglein-3 (GenBank accession number P32926). The fragments were cloned into pQE30 via the *BamH* I and *Kpn* I sites, and transformed into *E. coli* BL21-CodonPlus (DE3)-RIPL with pREP4 repressor plasmid. N-terminal His-tagged desmogleins were purified using a QIAexpress protein purification system (Qiagen), as previously described (Nakata et al., 2011).

### Analysis of *S. pyogenes* supernatant-induced cleavage of desmosomal proteins

Overnight cultures of *S. pyogenes* clinical isolates were centrifuged at 7000 × g for 5 min, then 20 μl of each culture supernatant was incubated with 3 μg of Dsg1 or Dsg3 at 37°C for 3 h. To search for a bacterial protease that cleaves desmogleins, culture supernatants were individually pretreated at room temperature for 30 min with the following protease inhibitors; *N*-ethylmaleimide (1 mM), E-64 (10 μM), AEBSF (1 mM), benzamidine HCl (1 mM), pepstatin (1.5 μM), bestatin (1 mM), and EDTA (1 mM). All protease inhibitors were purchased from Sigma-Aldrich. Cleavage of desmogleins was detected by western blot analysis with specific antibodies against human desmoglein-1 (mouse mAb, R&D Systems) or human desmoglein-3 (mouse mAb, R&D Systems). A horseradish peroxidase (HRP)-conjugated antibody against mouse IgG (Cell Signaling) was used as the secondary antibody. Immunoreactive bands were detected using Pierce ECL Western Blotting Substrate (Thermo Fisher Scientific).

### Murine model of epicutaneous infection with *S. pyogenes*

Mouse experiments were performed using a previously reported epicutaneous infection model, with minor modifications (Nakamura et al., 2013). Briefly, *S. pyogenes* strains were grown to the mid-exponential phase (OD_600_ = 0.4), then washed with and resuspended in PBS. Dorsal skin of 6- to 8-week-old female C57BL/6 mice (Japan SLC, Inc.) was depilated 2 days before infection. A bacterial suspension (5 × 10^8^ CFU in 100 μl PBS) was placed on a 1 × 1 cm patch of sterile gauze, which is secured to the shaved skin with a transparent bio-occlusive dressing. At 3 days post-infection, cutaneous tissue was excised for histopathologic and quantitative analyses. The severity of cutaneous lesions was examined by two clinicians blinded to the experimental grouping. Pathological features, including erythema, edema, erosion, and purulence, were graded as follows: 0 (none), 1 (mild), 2 (moderate), and 3 (severe). Disease score was defined as the sum of the individual pathological scores. All mouse experiments were conducted under a protocol approved by the Animal Care and Use Committee of Osaka University Graduate School of Dentistry (Authorization number: 24-025-2).

### Histopathologic and immunohistochemistry studies

Cutaneous tissue samples were obtained and fixed with formalin, then embedded in paraffin and sectioned, and subjected to hematoxylin and eosin (HE) staining. Stained tissue sections were examined with an Olympus CX41 light microscope (Olympus) and images were captured using a Nikon Coolpix P340 digital camera (Nikon). The numbers of neutrophils infiltrated into the epidermis and dermis were enumerated according to cell morphology. For immunohistochemistry (IHC) staining, sections were subjected to labeling with primary antibodies targeting Group A carbohydrate (Abcam), desmoglein-1 (Abcam), and desmoglein-3 (Bioss Antibodies). After washing, the tissue sections were incubated with Alexa Fluor 488-conjugated anti-goat IgG (Life Technologies) or Alexa Fluor 647-conjugated anti-rabbit IgG (Thermo Fisher Scientific), followed by staining with Hoechst 33342 (Thermo Fisher Scientific). Imaging was performed using a Zeiss Axio Observer D1 (Carl Zeiss) and analyzed with AxioVision software.

### Statistical analysis

Statistical differences for severity of cutaneous lesions, bacterial association, and neutrophil infiltration among the groups infected with the various *S. pyogenes* strains were examined using one-way ANOVA, followed by Tukey's multiple comparison test. All analyses were performed using GraphPad Prism (version 7.03; GraphPad Software). A confidence interval with a *p*-value of <0.05 was considered to be significant.

## Results

### *S. pyogenes* culture supernatant induces cleavage of Dsg1 and Dsg3

**Figure 1:**
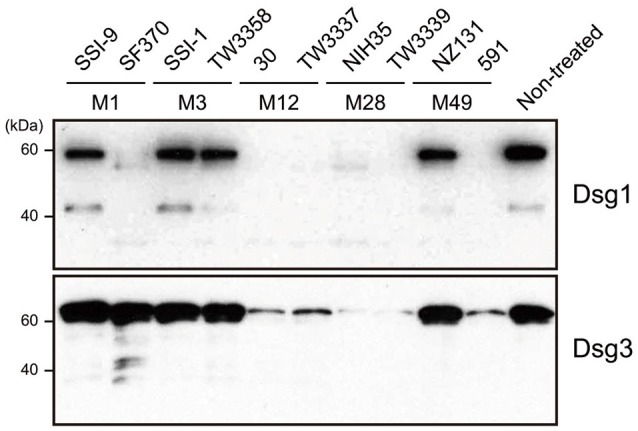
*S. pyogenes* culture supernatants induce cleavage of Dsg1 and Dsg3. Recombinant extracellular domains of Dsg1 and Dsg3 were separately incubated with culture supernatants from *S. pyogenes* clinical isolates for 3 h at 37°C. Sample proteins were separated by SDS-PAGE under a reducing condition, and subjected to western blot analysis using specific antibodies against the extracellular domain of Dsg1 and Dsg3.

The extracellular domain of desmogleins, which is composed of a series of highly conserved extracellular repeats (EC1-4) and a more variable extracellular anchor, is crucial for cell-cell adhesion (Delva et al., 2009). To examine whether *S. pyogenes* causes dysfunction of DMs, culture supernatants from strains with various serotypes recovered from both invasive (strains SSI-9, SSI-1, 30, NIH35) and non-invasive (strains SF370, TW3358, TW3337, TW3339, NZ131, 591) diseases were incubated with the extracellular domain of human Dsg1 or Dsg3, then cleavage of desmogleins was analyzed using western blot analysis (Figure 1). Intact forms with an apparent molecular mass of ~60 kDa were detected with antibodies against the extracellular domain of Dsg1 or Dsg3. Incubation with culture supernatants of strains 30, TW3337, NIH35, TW3339, and 591, but not in those from cultures of strains SSI-9, SSI-1, TW3358, and NZ131, indicated loss of the band corresponding to Dsg1 and Dsg3. Culture supernatant of SF370 cleaved the Dsg1 fragment, whereas only a few ladder bands reflecting cleavage products were detected in case of Dsg3. Notably, cleavage of both Dsg1 and Dsg3 was abolished by pretreatment with a protease inhibitor mixture (data not shown). These results suggest that several *S. pyogenes* strains secrete a protease responsible for the ectodomain shedding of Dsg1 and Dsg3.

### Streptococcal cysteine protease is a bacterial determinant for cleavage of desmogleins

**Figure 2:**
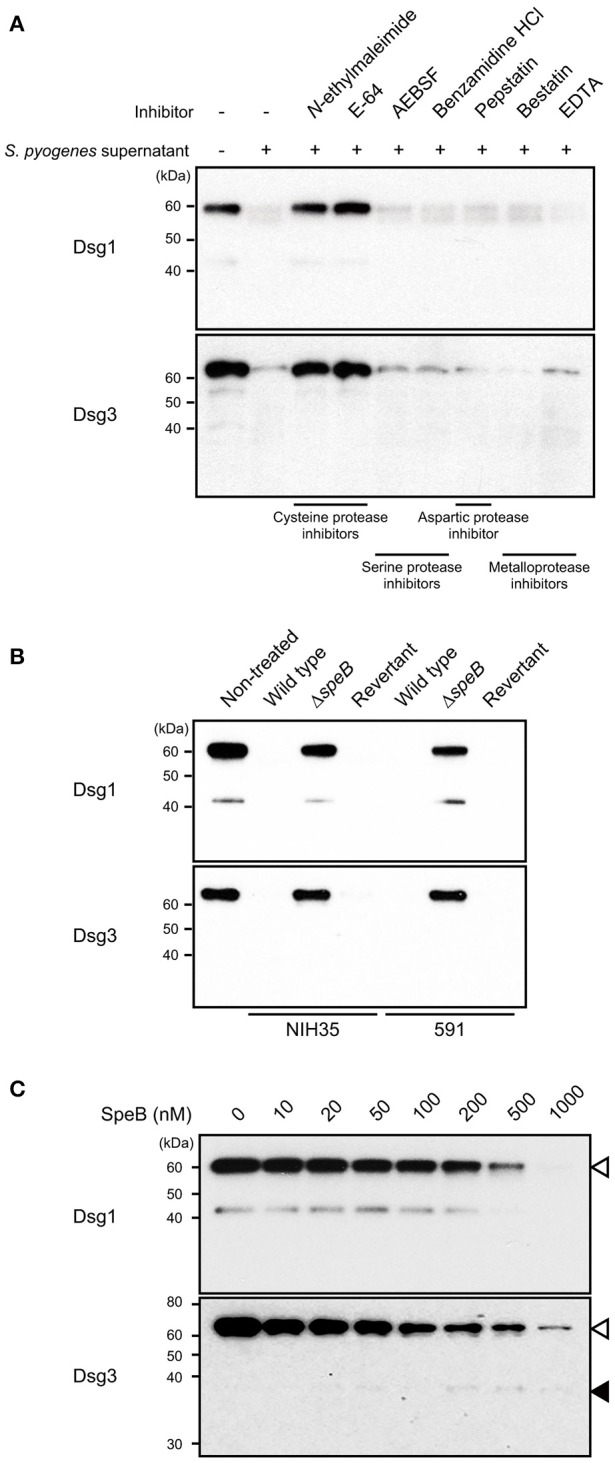
SpeB is a bacterial determinant for cleavage of desmogleins. **(A)** Culture supernatant from *S. pyogenes* strain 591 was pretreated with various types of protease inhibitors at room temperature for 30 min, then incubated with Dsg1 or Dsg3 recombinant protein at 37°C for 3 h. **(B)** Recombinant desmogleins were separately treated with culture supernatants from *S. pyogenes* strains at 37°C for 3 h. An in-frame *speB* deletion mutant and its revertant strain with a background of NIH35 or 591 were employed for analysis. **(C)** Dsg1 and Dsg3 recombinant proteins were separately incubated with various concentrations of recombinant SpeB at 37°C for 3 h, then cleavage of desmogleins was detected by western blot analysis. White and black arrowheads indicate the full-length band and cleavage fragment, respectively.

To screen for a bacterial protease that cleaves desmogleins, culture supernatant from strain 591, a prominent degrader isolated from a case of skin infection, was pre-incubated with several types of protease inhibitors, followed by incubation with extracellular fragments of Dsg1 or Dsg3 (Figure 2A). Culture supernatant-induced cleavage of Dsg1 and Dsg3 was completely abrogated by pretreatment with cysteine protease inhibitors, such as *N*-ethylmaleimide and E-64. In contrast, protease inhibitors targeting serine protease, aspartic protease, and metalloprotease did not have effects on desmoglein degradation. These findings indicate that cysteine protease is involved in cleavage of both Dsg1 and Dsg3.

*S. pyogenes* secretes two kinds of cysteine protease, streptococcal pyrogenic exotoxin B (SpeB) and an immunoglobulin G-degrading enzyme (Ides or Mac-1). Since E-64 was reported to have effects on the proteolytic activity of SpeB but not of IdeS, involvement of SpeB in the cleavage of Dsg1 and Dsg3 was examined in the following experiments (Von Pawel-Rammingen et al., 2002).

SpeB, the predominant extracellular protein found in *S. pyogenes* culture supernatants, degrades the host extracellular matrix, immunoglobulins, complement components, and even streptococcal surface proteins (Chiang-Ni and Wu, 2008; Terao et al., 2008; Nelson et al., 2011; Honda-Ogawa et al., 2013). Hence, an in-frame *speB* deletion mutant and its revertant strain with a background of NIH35 or 591 were employed to verify whether SpeB is a determinant for cleavage of desmogleins. Growth rates of the mutant and revertant strains were nearly identical to that of the wild type (data not shown). Proteolytic activity against Dsg1 and Dsg3 was abolished by mutagenesis of *speB*, whereas it was completely restored in the revertant strain (Figure 2B). Next, a direct interaction between SpeB and desmogleins was assessed using recombinant SpeB. Loss of intact bands corresponding to the extracellular domains of Dsg1 or Dsg3 was detected in an SpeB concentration-dependent manner (Figure 2C). We also detected an ~43-kDa band in Dsg1 samples treated with 10–200 nM of SpeB. Since this fragment was found even in the non-treated samples and disappeared in the presence of high concentrations of SpeB, it seems to be a naturally occurring degradation product of Dsg1. Meanwhile, a weak band with an apparent molecular mass of 37 kDa, reflecting a cleavage product, was observed in the Dsg3 samples treated with 200–1000 nM of SpeB. Despite the decrease in intensity of the intact band, distinct cleavage products were not detected in either the Dsg1 or Dsg3 samples, even when treated with 1000 nM SpeB. These findings suggest that SpeB released from *S. pyogenes* targets several sites of extracellular Dsg1 and Dsg3, and degrades those to low molecular mass fragments.

Notably, our previous findings of high levels of SpeB activity detected in culture supernatants from strains 30, TW3337, NIH35, TW3339, and 591 were correlated to the ability of the strain to cleave desmogleins (Sumitomo et al., 2013). Although the SpeB activity of strain SF370 was relatively low, marked cleavage of Dsg1 was detected (Figure 1). To examine whether SpeB proteolytic activity depends on sequence variations of SpeB, the SpeB DNA sequences of the tested strains were either sequenced or extracted from genome databases. Thus far, three major SpeB variants have been reported (Stockbauer et al., 1999). As previously demonstrated, we found that SpeB type was well-correlated with *emm* type, though sequence variations had no apparent association with proteolytic activity against desmogleins (data not shown). Thus, an alternative protease specific for Dsg1 was likely involved in cleavage of desmoglein by the culture supernatant of SF370. Taken together, our results indicate that the proteolytic activity of SpeB is a predominant determinant for *S. pyogenes*-mediated cleavage of desmogleins.

### SpeB-mediated dysfunction of DMs contributes to development of cutaneous lesions

**Figure 3:**
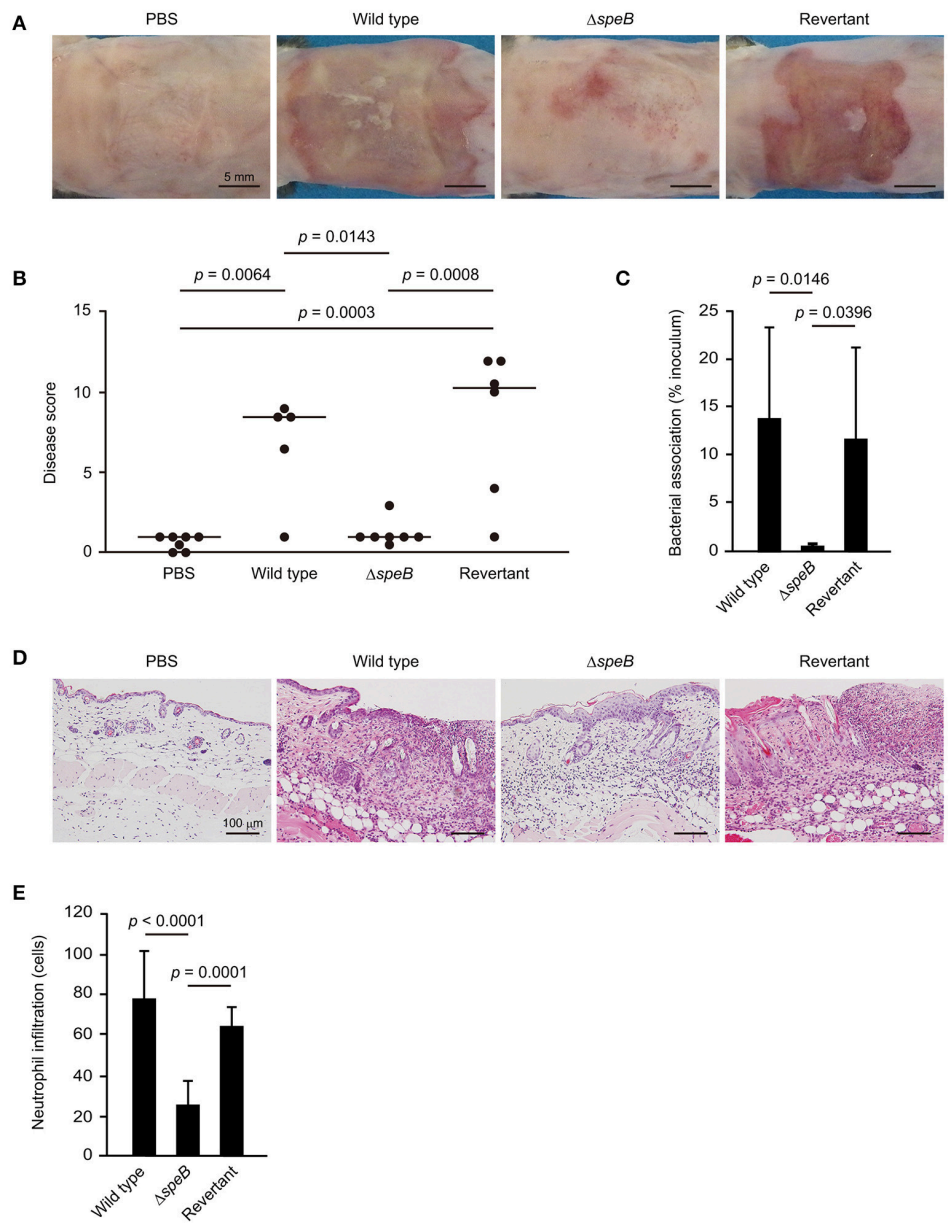
SpeB is critical for the development of cutaneous lesions. Mice were infected in an epicutaneous manner with strain 591, the *speB* deletion mutant, or the revertant strain for 3 days. **(A)** Representative gross appearance of mouse skin samples after infection with the *S. pyogenes* strains. **(B)** Cutaneous disease score was determined as the sum of individual scores for erythema, edema, erosion, and purulence, graded as follows: 0 (none), 1 (mild), 2 (moderate), and 3 (severe). The median value for each group is shown as a horizontal bar. **(C)** Each cutaneous tissue homogenate was serially diluted and plated on a THY agar plate containing 5% sheep blood. Data shown represent the mean ± S.D. of quintuplet samples and are representative of at least three independent experiments. **(D)** Cutaneous tissues from infection sites were stained with hematoxylin and eosin. Data shown are representative of at least three separate experiments. **(E)** Cutaneous inflammation was evaluated based on neutrophil infiltration into the epidermis and dermis. Data were obtained from five random fields of view (x400) and are presented as the mean ± S.D. of 9 independent samples. Statistically significant differences were evaluated using one-way ANOVA, followed by Tukey's multiple comparison test.

The sequences of desmogleins have been demonstrated to be highly conserved among different mammalian species at the protein level (Mahoney et al., 2002). Based on our *in vitro* observation that SpeB degrades DM components, a mouse epicutaneous infection model was utilized to examine whether SpeB is involved in cutaneous infection development. At 3 days following infection, mice infected with the wild-type as well as those with the revertant strains developed skin lesions, which were accompanied by local erythema, edema, erosion, and purulence (Figure 3A, Supplementary Figure 1). In contrast, only minimal inflammation and no lesion formation was observed in mice infected with the *speB* mutant. Based on gross pathological findings (Supplementary Figure 1), the severity of cutaneous lesions was evaluated by disease score, which was determined as the sum of individual pathological scores for erythema, edema, erosion, and purulence. Mice infected with the wild-type strain had high cutaneous lesion scores, whereas development of those lesions was significantly repressed by mutagenesis of the *speB* gene and restored by complementation (Figure 3B). Consistent with the severity of cutaneous lesions, a significant decrease in bacterial association was detected in the skin of mice infected with the *speB* mutant, as compared with those infected with the wild-type or revertant strains (Figure 3C). Histological findings revealed extensive ulcerative lesions with abundant bacteria and nuclear dust in the skin of mice infected with the wild-type strain, even in the absence of wounding (Figure 3D). Moreover, areas of spongiosis adjacent to the ulcerative lesions in the epidermis were observed, likely a consequence of epidermal transversion of numerous inflammatory cells accompanied by fluid accumulation. A large number of neutrophil-rich inflammatory infiltrates was also found not only in subcutaneous tissue and the deep dermis layer, but also in the superficial dermal layer adjoining the areas of ulceration (Figures 3D,E). In contrast, host inflammatory response was limited to the subcutis and deep dermal layers in mice infected with the *speB* mutant. Although mutagenesis of the *speB* gene abolished the ability of *S. pyogenes* to form ulceration, marked epidermal hyperplasia developed around the sites of infection. Histopathology findings of mice infected with the revertant strain were nearly identical to those of mice infected with the wild type.

**Figure 4:**
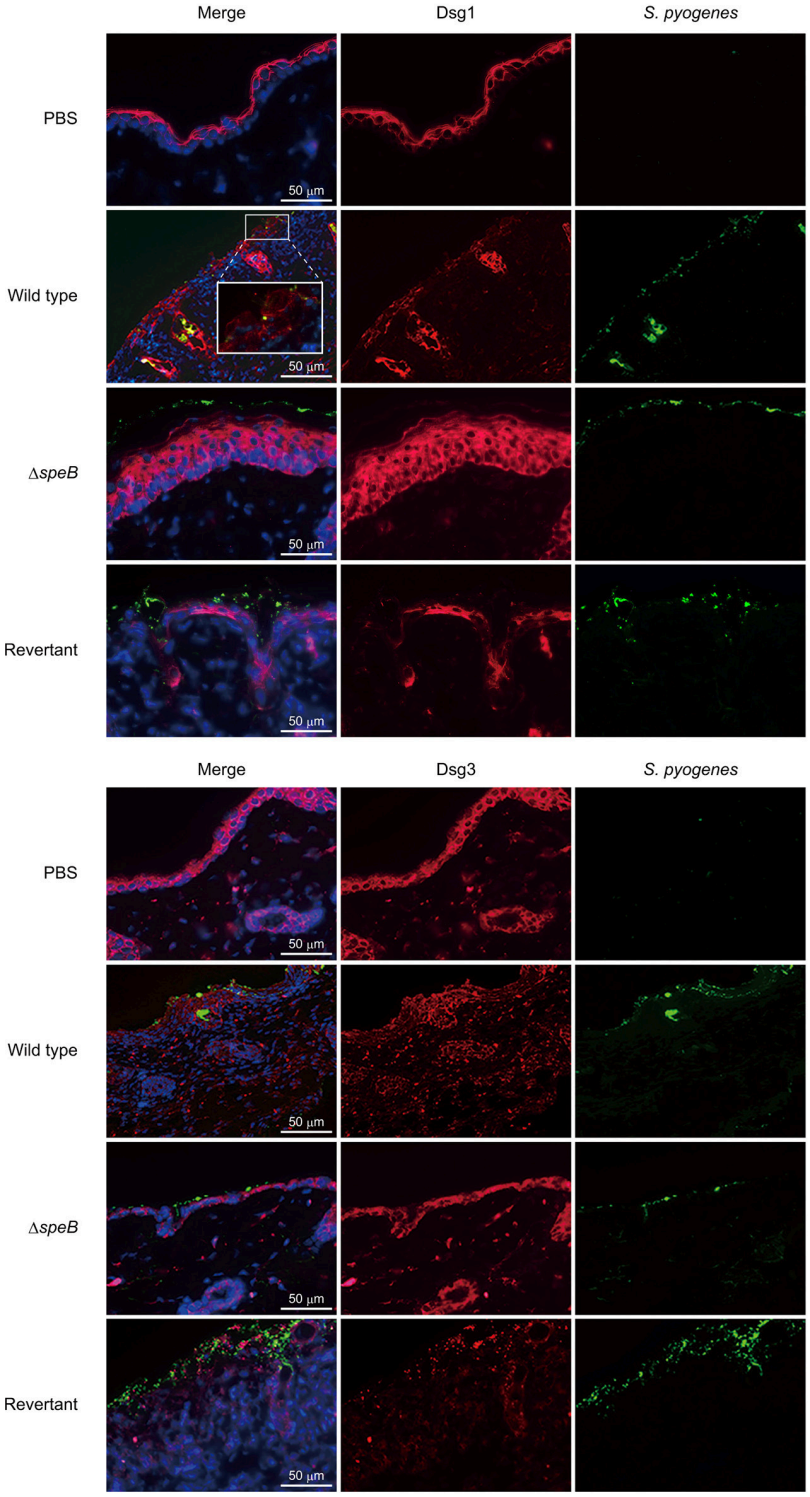
SpeB-mediated cleavage of desmogleins contributes to epidermal barrier dysfunction. Cutaneous sections obtained from mice infected with the examined *S. pyogenes* strains were subjected to immunofluorescence staining. Dsg1 and Dsg3 were labeled with anti-Dsg1 and anti-Dsg3 antibodies, respectively, followed by incubation with an Alexa Fluor 647-conjugated antibody. *S. pyogenes* was labeled with anti-Group A carbohydrate and Alexa Fluor 488-conjugated antibodies, and cell nuclei were stained with Hoechst 33342. Obtained tissue sections were analyzed using a confocal laser microscope. The boxed area is magnified in the box panel below. Data shown are representative of at least three separate experiments.

To clarify the correlation between the barrier function of DMs and cutaneous infection, skin sections were subjected to IHC analysis. Although typical desmoglein expression was observed in the PBS-treated samples, fluorescent intensity for both Dsg1 and Dsg3 was markedly diminished in the skin of mice infected with the wild-type as well as the revertant strains (Figure 4). Notably, bacterial cells were found translocated into subepidermal tissues through cleaved Dsg1 sites. On the other hand, bacterial distribution of the *speB* mutant was restricted to the keratinocyte surface in epidermis with retained Dsg1 and Dsg3. Consistent with the histological findings, marked epidermal hyperplasia, shown by staining with an anti-Dsg1 antibody, was observed in tissues infected with the *speB* mutant. Based on the results of *in vivo* experiments with the present epicutaneous infection model, we concluded that SpeB is a crucial bacterial factor for development of cutaneous lesions induced by *S. pyogenes* infection, accompanied by destruction of the desmosomal structure associated with the epidermal barrier.

## Discussion

Impetigo, the most common type of cutaneous infection, is classified as non-bullous and bullous impetigo. Patients with non-bullous impetigo, which accounts for more than 70% of reported cases, are characterized by a honey-colored crust on the face or extremities. The etiological agents of non-bullous impetigo are *S. pyogenes* and *Staphylococcus aureus*, either separately or in combination. The less common bullous impetigo, which is caused exclusively by *S. aureus*, results in fragile fluid-filled vesicles and blisters. Exfoliative toxins (ETs) produced by *S. aureus* have been identified as a determinant for dissociation of epidermal cells with blister formation in bullous impetigo, while the pathogenesis mechanisms of the non-bullous form remain to be elucidated (Amagai et al., 2000). Despite the fact that *S. aureus* is the predominant causative organism in both diseases, clinical and histopathological manifestations of non-bullous impetigo are distinct from those of bullous impetigo (Empinotti et al., 2012; Pereira, 2014). Thus, we speculated that bacterial factors produced by *S. pyogenes* are critical for development of acute pyoderma in cases of non-bullous impetigo.

The stratum corneum functions as a physical barrier for skin, while nucleated epidermis is also associated with epidermal integrity through the functions of TJs, AJs, and DMs. In bullous impetigo, staphylococcal serine protease ETs cleave a single peptide bond following a glutamic acid residue at position 381 within extracellular domain 3 (EC3) of Dsg1, whereas there is no specificity for closely homologous proteins such as Dsg3 and E-cadherin (Hanakawa et al., 2002). ET-mediated cleavage of Dsg1 results in loss of adhesion between neighboring keratinocytes, leading to formation of blisters within the superficial layer of the epidermis (Amagai et al., 2000). The present findings are the first reported obvious evidence that SpeB secreted from *S. pyogenes* cleaves not only Dsg1 but also Dsg3. Desmogleins are members of the cadherin family that anchor adjacent epithelial cells to one another in DMs through their EC domains, and play crucial roles in maintaining the structure and barrier function of the epidermis (Delva et al., 2009). The EC region is constituted of 4 cadherin repeats with calcium-binding motifs conserved among all members of the cadherin family, including desmogleins and E-cadherin. In addition to DM constituents, our previous study demonstrated that components of TJs and AJs, such as occluidin and E-cadherin, are targets of SpeB (Sumitomo et al., 2013). Indeed, SpeB directly cleaves E-cadherin at the region neighboring conserved calcium-binding motifs between each EC domain of E-cadherin, which leads to destabilization of the epithelial barrier. The present findings indicate that the substrate specificity of causative proteases may be related to the distinct pathogenesis of impetigo.

Skin colonization with *S. pyogenes* is considered to be one of the predisposing factors for non-bullous impetigo. A murine subcutaneous infection model is widely used as an animal model for investigation of *S. pyogenes* superficial skin infections (Bunce et al., 1992). Although multiple bacterial factors including SpeB have been demonstrated to be associated with the pathogenesis, this model, in which bacteria are injected into skin, is not designed to precisely mimic the mode of topical infection encountered under natural conditions. Animal models used for examinations of specific human pathogens, including *S. pyogenes*, frequently fail to mimic key aspects of the infectious process. Indeed, mice are thought to be an unsuitable animal model of *S. pyogenes* cutaneous infection because of histological differences between human and murine epidermal tissues. Scaramuzzino and coworkers generated an innovative humanized mouse model with transplantation of human neonatal foreskin onto immunocompromised SCID mice and presented results showing that SpeB plays a critical role in development of cutaneous lesions during the early stages of infection (Scaramuzzino et al., 2000; Svensson et al., 2000). Although this model precisely reflects key episodes occurring within human hosts, both surgical skill and a source of applicable human tissues are required for construction of the model. In the present study, we used a modified mouse epicutaneous infection model to examine whether SpeB-mediated cleavage of junctional proteins is associated with both bacterial invasion and development of impetigo lesions (Nakamura et al., 2013).

Formation of subcorneal pustules, a typical feature of non-bullous impetigo, was not observed in the present mouse model. Notably, even in the absence of wounding, ulcerative lesions with abundant bacterial and neutrophil infiltrations were found developed in the epidermal and superficial dermal layers of skin of mice infected with the wild-type strain, which closely resembled histopathological features seen in ecthyma, a severe form of impetigo (Empinotti et al., 2012). However, a high-dose bacterial inoculation was a prerequisite for development of demonstrable cutaneous lesions. Indeed, studies using human skin-engrafted SCID mice and a M33 strain have presented evidence showing that low-dose bacterial inoculation (<10^3^ CFU) results in formation of subcorneal pustules (Scaramuzzino et al., 2000; Svensson et al., 2000). Thus, the histological differences between human and murine epidermal tissues, including thickness of the epidermal layer, together with differences in virulence of tested strains, were correlated with the absence of impetigo-like lesions in our model. Spongiosis was also noted adjacent to ulcerative lesions in the epidermis. Therefore, the proteolytic activities of SpeB, epidermal transversion of numerous inflammatory cells and/or the combination of both factors, are assumed to be critical for formation of spongiosis in epidermis infected with *S. pyogenes*. This speculation is supported by our observation that abrogation of pronounced spongiosis accompanied by neutrophil-rich inflammatory infiltrates in the skin of mice infected with *S. pyogenes* with *speB* deletion. In our previous experiments with an *in vitro* model of the epithelial barrier, we found that SpeB-mediated cleavage of intercellular junctions allowed bacterial translocation via a paracellular route (Sumitomo et al., 2013). Confirming the findings of another previous study, bacterial translocation was found to occur in impaired intercellular junctions in the present epicutaneous mouse model, as shown by labeling with anti-Dsg1 and anti-Dsg3 antibodies. Together, our findings emphasize that SpeB-induced dysfunction of the epidermal barrier allows bacterial invasion into deeper tissues, leading to development of ecthyma-like lesions caused by *S. pyogenes* infection. In addition to junctional proteins, SpeB has been shown to degrade extracellular matrix proteins, immunoglobulins, ckemokines, and complement components (Chiang-Ni and Wu, 2008; Terao et al., 2008; Nelson et al., 2011; Honda-Ogawa et al., 2013). It is possible that adverse events caused by the broad spectrum proteolytic activity, including perturbation of immune system, may be also related to destabilization of the epidermal barrier.

The present histopathological results of mouse skin infected with the *speB* mutant revealed marked keratinocyte hyperproliferation with inflammatory infiltrates, a typical phenotype observed in patients with inflammatory cutaneous diseases such as psoriasis (Park et al., 2009; Valdimarsson et al., 2009). Similarly, development of acanthosis, which reflects detachment of granular keratinocytes, in immunocompromised SCID mice infected with an *speB* mutant strain has been reported (Svensson et al., 2000). Psoriasis is an immune-mediated chronic cutaneous disorder characterized by epidermal hyperplasia with acanthosis and focal parakeratosis. High levels of serum Th17-driven cytokines, including IL-17A and IL-23, seen in affected patients are associated with epidermal hyperplasia and quick spread of a guttate type morphology (Choe et al., 2012; Yilmaz et al., 2012). Streptococcal pharyngitis precedes development of psoriasis, especially guttate psoriasis, contributing to exacerbation of lesions in HLA-Cw6^+^ patients (Telfer et al., 1992; Gudjonsson et al., 2006). Indeed, an *S. pyogenes*-mediated dominant Th17 response via cutaneous lymphocyte antigen-positive T cell-dependent epidermal cell activation has been implicated in the features of keratinocytes in psoriasis, such as IL-17 production, and decreased filaggrin and loricrin expression (Ruiz-Romeu et al., 2016). Although Th17-related cytokines are not considered to be main players in the pathogenesis of acute cutaneous *S. pyogenes* infection, inflammatory response mediated through streptococcal factors other than SpeB might be related to development of epidermal hyperplasia.

SpeB has been implicated in establishment of localized *S. pyogenes* skin infections, while its precise role in the pathogenesis of invasive disease remains unclear (Cole et al., 2006). We also noted that SpeB-associated protease activity was correlated with cleavage of intercellular junctions, whereas it was not associated with disease severity related to *S. pyogenes* isolates derived from invasive or non-invasive infections. Destabilization of the cell-cell junction has been proposed as a prerequisite for not only development of purulent lesions at primary sites of infection, such as the pharynx and skin, but also for deeper bacterial invasion of tissue at the initial stage of an invasive infection. Although the SpeB activities of strains SSI-9 and SSI-1, isolated from invasive disease, were relatively low, alternative factors, including streptolysin S, streptokinase, and the capsule, are thought to be involved in barrier destabilization (Cywes and Wessels, 2001; Sumitomo et al., 2011). The CovRS (also termed CsrRS) two-component system and Rgg (also termed RopB) are considered to be transcriptional regulators of SpeB (Chaussee et al., 1999; Heath et al., 1999), and several investigations have indicated that the transition from local to systemic infection is related to spontaneous mutations of the *covRS* and *rgg* genes (Hollands et al., 2008; Ikebe et al., 2010; Carroll et al., 2011). Since these mutations are thought to occur under innate immune selection, SpeB activity might be changed under *in vivo* conditions. Our findings also support the notion that SpeB is a crucial determinant for establishment of a localized *S. pyogenes* infection, though the correlation of SpeB expression with disease severity remains controversial.

Together with our previous *in vitro* observations, the present *in vivo* findings indicate that the streptococcal cysteine protease SpeB cleaves not only DMs proteins, but also components of TJs and AJs, thus contributing to development of ecthyma-like lesions, a severe form of impetigo. SpeB-induced opening of epidermal junctions may be sufficient to allow bacterial translocation via a paracellular route, thereby leading to severe clinical manifestations in patients with an *S. pyogenes* cutaneous infection. Elucidation of the novel pathophysiological aspects of SpeB will be beneficial for development of new strategies to prevent and manage cutaneous *S. pyogenes*-associated infection.

## Author contributions

TS and SK conceived and designed the experiments. TS, YM, YN, MH-O, SN, MY, HM, YT, and MN performed the experiments and analyzed the data. TS, MN, and SK contributed to the writing of the manuscript. All authors participated in discussions of the research and reviewed the manuscript.

### Conflict of interest statement

The authors declare that the research was conducted in the absence of any commercial or financial relationships that could be construed as a potential conflict of interest.

## Supplementary material

Figure 1 Mice were infected in an epicutaneous manner with strain 591, an *speB* deletion mutant, or a revertant strain for 3 days. Disease severity for pathological features, such as **(A)** erythema, **(B)** edema, **(C)** erosion, and **(D)** purulence, was scored as follows: 0 (none), 1 (mild), 2 (moderate), and 3 (severe). The median value for each group is shown as a horizontal bar. Statistically significant differences were evaluated using one-way ANOVA, followed by Tukey's multiple comparison test.
