# Supplementary figures and images for: Streptococcal Cysteine Protease-Mediated Cleavage of Desmogleins Is Involved in the Pathogenesis of Cutaneous Infection
Source: Front Cell Infect Microbiol. 2018 Jan 24;8:10. doi: 10.3389/fcimb.2018.00010 (PMC5787553; doi:10.3389/fcimb.2018.00010)

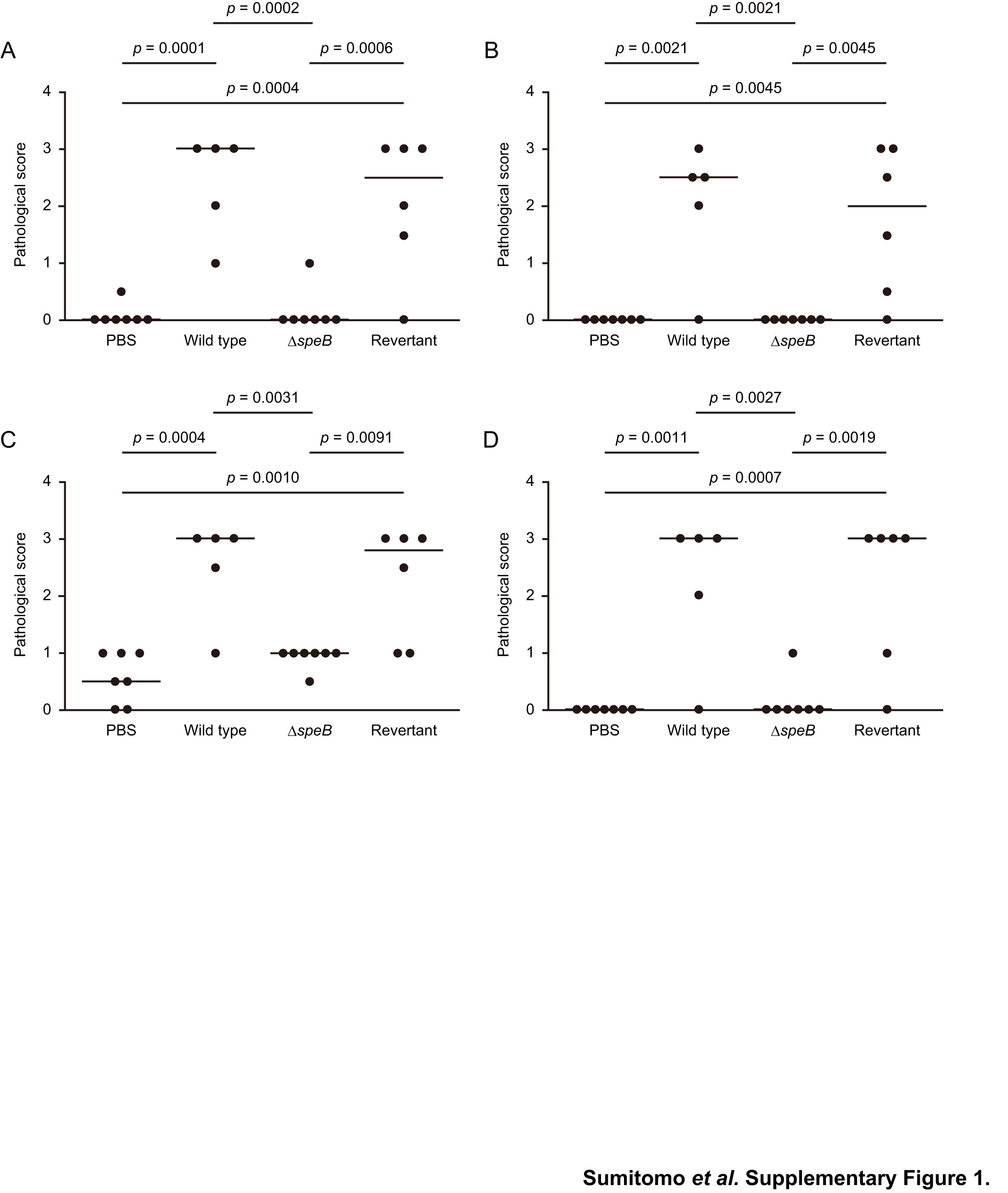

Supplement: Figure 1 — Mice were infected in an epicutaneous manner with strain 591, an speB deletion mutant, or a revertant strain for 3 days. Disease severity for pathological features, such as (A) erythema, (B) edema, (C) erosion, and (D) purulence, was scored as follows: 0 (none), 1 (mild), 2 (moderate), and 3 (severe). The median value for each group is shown as a horizontal bar. Statistically significant differences were evaluated using one-way ANOVA, followed by Tukey's multiple comparison test. [file Image1.JPEG]
